# Supplementary material for: Structural Variation Detection and Association Analysis of Whole-Genome-Sequence Data from 16,905 Alzheimer’s Diseases Sequencing Project Subjects
Source: Res Sq. 2023 Oct 5:rs.3.rs-3353179. Preprint. [Version 1] doi: 10.21203/rs.3.rs-3353179/v1 (PMC10602095; doi:10.21203/rs.3.rs-3353179/v1)
Supplement: Supplement 1 [file NIHPPRS3353179V1-supplement-1.pdf]

Supplementary Files

This is a list of supplementary files associated with this preprint. Click to download.

- SupplementaryFigures.docx
- SupplementaryTables.xlsx
